# Gene-Based Therapeutics for Inherited Retinal Diseases

**DOI:** 10.3389/fgene.2021.794805

**Published:** 2022-01-07

**Authors:** Beau J. Fenner, Tien-En Tan, Amutha Veluchamy Barathi, Sai Bo Bo Tun, Sia Wey Yeo, Andrew S. H. Tsai, Shu Yen Lee, Chui Ming Gemmy Cheung, Choi Mun Chan, Jodhbir S. Mehta, Kelvin Y. C. Teo

**Affiliations:** ^1^ Singapore National Eye Centre, Singapore, Singapore; ^2^ Singapore Eye Research Institute, Singapore, Singapore; ^3^ Duke-NUS Graduate Medical School, Ophthalmology and Visual Sciences Academic Clinical Programme, Singapore, Singapore; ^4^ School of Material Science and Engineering, Nanyang Technological University, Singapore, Singapore; ^5^ Yong Loo Lin School of Medicine, Department of Ophthalmology, National University of Singapore, Singapore, Singapore

**Keywords:** inherited retinal diseases (IRDs), gene tharapy, adeno-associated virus, RNA editing, CRISPR, optogenetic, antisense oligonucleotides, retina

## Abstract

Inherited retinal diseases (IRDs) are a heterogenous group of orphan eye diseases that typically result from monogenic mutations and are considered attractive targets for gene-based therapeutics. Following the approval of an IRD gene replacement therapy for Leber’s congenital amaurosis due to *RPE65* mutations, there has been an intensive international research effort to identify the optimal gene therapy approaches for a range of IRDs and many are now undergoing clinical trials. In this review we explore therapeutic challenges posed by IRDs and review current and future approaches that may be applicable to different subsets of IRD mutations. Emphasis is placed on five distinct approaches to gene-based therapy that have potential to treat the full spectrum of IRDs: 1) gene replacement using adeno-associated virus (AAV) and nonviral delivery vectors, 2) genome editing via the CRISPR/Cas9 system, 3) RNA editing by endogenous and exogenous ADAR, 4) mRNA targeting with antisense oligonucleotides for gene knockdown and splicing modification, and 5) optogenetic approaches that aim to replace the function of native retinal photoreceptors by engineering other retinal cell types to become capable of phototransduction.

## Introduction

Improvements to global living standards and increased life expectancies over the past century have led to a transition in medical research to noninfectious diseases (COVID19 notwithstanding) and disabilities that impair quality of life. Visual impairment represents a special disability that has profound impact on a person’s ability to interact with the world and has been identified as an area of focus by the World Health Organization (Pizzarello et al., 2004; Bourne, 2020). Recent epidemiological analyses indicate that while major causes of blindness such as cataract, undercorrected refractive error, age-related macular degeneration, glaucoma, and diabetic retinopathy are starting to decline in prevalence, these conditions predominantly affect older age groups (GBD 2019 Blindness and Vision Impairment Collaborators and Vision Loss Expert Group of the Global Burden of Disease Study, 2021). In contrast, blindness among working age adults, which was previously due mainly to diabetic retinopathy in developed nations, is now predominantly due to a very large and heterogenous group of blinding conditions collectively termed inherited retinal diseases (IRDs) (Liew et al., 2014; Heath Jeffery et al., 2021). IRDs have, until very recently, remained essentially untreatable.

IRDs are a heterogeneous group of orphan eye diseases with a prevalence estimated at between 0.06 and 0.2% (Galvin et al., 2020; Gong et al., 2021), with a global IRD caseload in the range of 5–10 million individuals (Hanany et al., 2020). Onset is highly variable, ranging from the first year of life all the way to late adulthood, and presenting symptoms can vary from nyctalopia and photosensitivity to profound vision loss with early-onset nystagmus. The societal burden of IRDs has been quantified in recent studies that investigated the socioeconomic impact of IRDs in the US and western Europe. Despite their scarcity, the societal cost of IRDs in the United Kingdom and United States were estimated at more than USD 700 million and USD 30 billion, respectively (Galvin et al., 2020; Gong et al., 2021). Much of this cost is presumably due to the profound impact that IRDs have on the economic productivity of working-age individuals, where improvements in community eye screening has seen IRDs overtake diabetic retinopathy as the leading cause of blindness among working age adults in some developed nations (Hanany et al., 2020; Heath Jeffery et al., 2021). Among Asian populations the spectrum of IRDs is similarly diverse, although IRD genotypes differ considerably from that in Western populations (Sen et al., 2008; Oishi et al., 2014; Arai et al., 2015; Wang et al., 2018; Yohe et al., 2020; Ma et al., 2021).

The phenotypic heterogeneity of IRDs is brought about by the diverse array of genotypes responsible for IRDs. Presently there are at least 270 discrete genetic loci responsible for IRDs in humans (RetNet www.sph.uth.edu/retnet), each of which may harbor many disease-causing variants with distinct clinical phenotypes, resulting in an almost overwhelming number of IRDs that can be encountered by an ophthalmologist. Despite this, the majority of IRDs can be classified as one of four broad subtypes: 1) rod-cone degenerations; 2) cone-rod degenerations; 3) chorioretinal degenerations; and 4) degenerations involving the macula, with the latter subtype often overlapping with the former subtypes. The clinical presentation of each subtype is usually consistent with the retinal cell types affected. Rod-cone degenerations, the most common of the IRDs, present with nyctalopia and peripheral visual field loss earlier in disease due to preferential degeneration of light-sensitive rods, followed by loss of central visual acuity and color vision later in the disease as cones become affected. Conversely, patients with cone-rod disease will initially present with reduced color vision, photophobia, or reduced visual acuity. Chorioretinal degenerations have variable presentation but typically present with nyctalopia and peripheral field loss with progression to central vision loss later in life. Degenerations involving the macula usually result in symptoms of metamorphopsia, reduced visual acuity, and progressive central and paracentral scotomata, while the peripheral vision is typically spared.

In addition to their ocular manifestations, many IRDs are associated with systemic disease, with the most well-known being Usher syndrome, an important cause of childhood sensorineural deafness (Saihan et al., 2009; Fuster-García et al., 2021). These cases are typically identified by internists and subsequently brought to attention of the ophthalmologist *via* routine referral for screening, but the ocular manifestations are occasionally the presenting complaint (Ehrenberg et al., 2019; Tatour and Ben-Yosef, 2020).

Progress in our understanding of IRDs at the genetic, biochemical, and cellular level was historically limited by their previously incurable nature. While many researchers spent decades assembling IRD cohorts and publishing phenotypic and genotypic findings for newly characterized IRDs, this was largely an academic pursuit. However, with the advent of high-throughput gene sequencing there has been a dramatic increase the rate of IRD gene discovery (Gao et al., 2019; Koyanagi et al., 2019; Holtan et al., 2020; Pontikos et al., 2020; Weisschuh et al., 2020; Qian et al., 2021). More recently, the pace of IRD research has further increased following the first FDA approval of gene therapy for an IRD (Russell et al., 2017).

In the current work, we review recent developments in our understanding of IRDs and their clinical outcomes before providing an outline of recent approaches taken to treat this important group in blinding retinal diseases.

## Pathophysiology of Inherited Retinal Diseases

**FIGURE 1:**
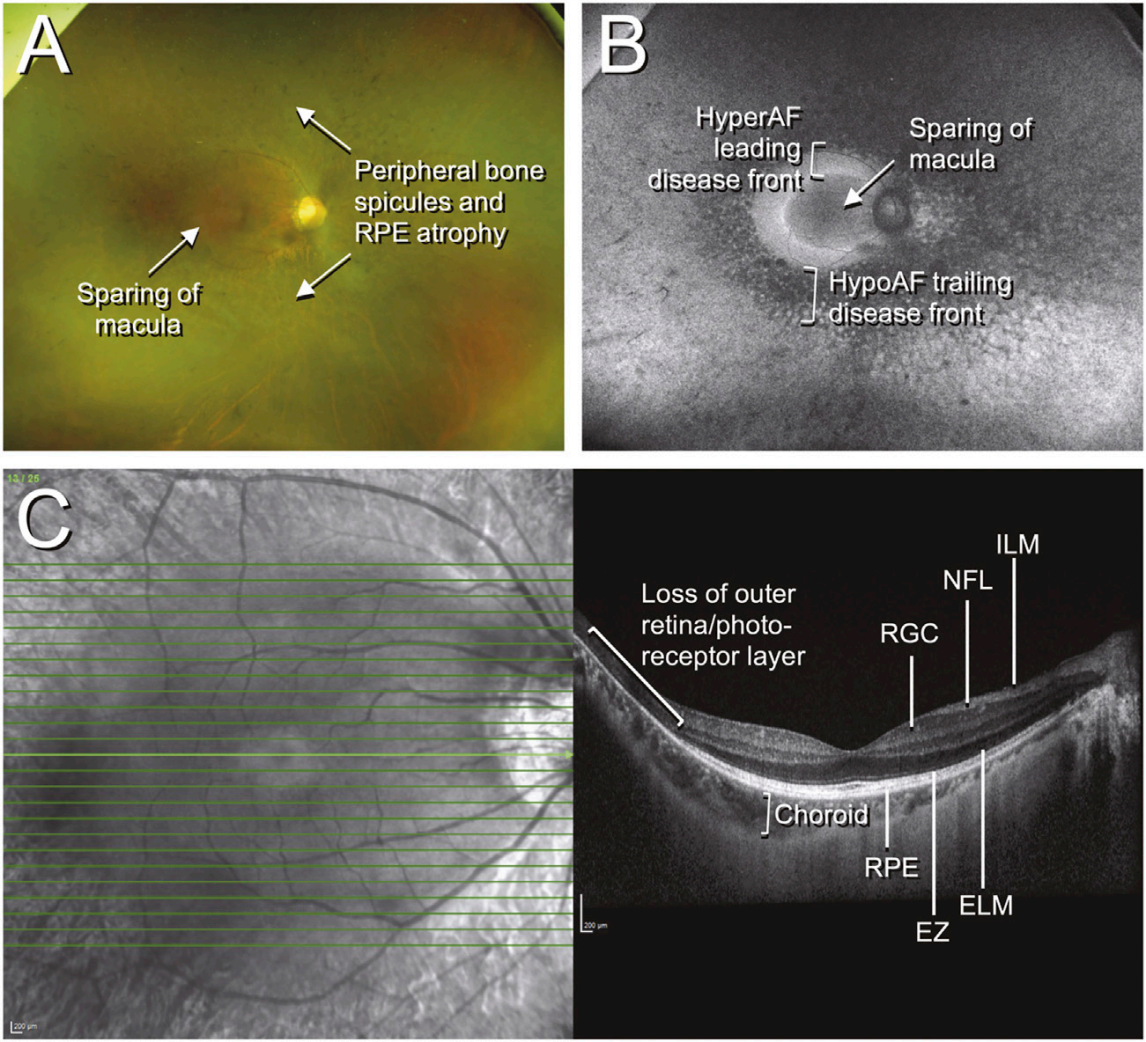
Color fundus photographs, autofluorescence, and optical coherence tomography (OCT) of retinitis pigmentosa. The patient was a 42-year-old Chinese male who presented with an incidental finding of peripheral field loss during an inpatient admission for migraine headache. A long-standing history of nyctalopia was elicited but there was no evidence of neurosensory hearing loss. Whole exome sequencing (WES) uncovered the presence of biallelic pathogenic *EYS* mutations. **(A)** Fundus imaging shows peripheral bone spicule-like pigmentary retinopathy with outer retinal atrophy and sparing of the macula; **(B)** fundus autofluorescence highlights the areas of disease, with the hypoautofluorescent (hypoAF) trailing disease front appearing as dark areas and the hyperautofluorescent (hyperAF) leading disease front appearing as brighter areas; **(C)** horizontal cross-sectional OCT at the level of the fovea shows intact retinal layers at the fovea with loss of the outer retina and photoreceptor layers in the periphery. Selected retinal layers relevant to gene-based therapy are shown: ILM, internal limiting membrane; NFL, nerve fiber layer; RGC, retinal ganglion cell layer; RPE, retinal pigment epithelium; EZ, ellipsoid zone (photoreceptor inner/outer segments); ELM, external limiting membrane.

**FIGURE 2:**
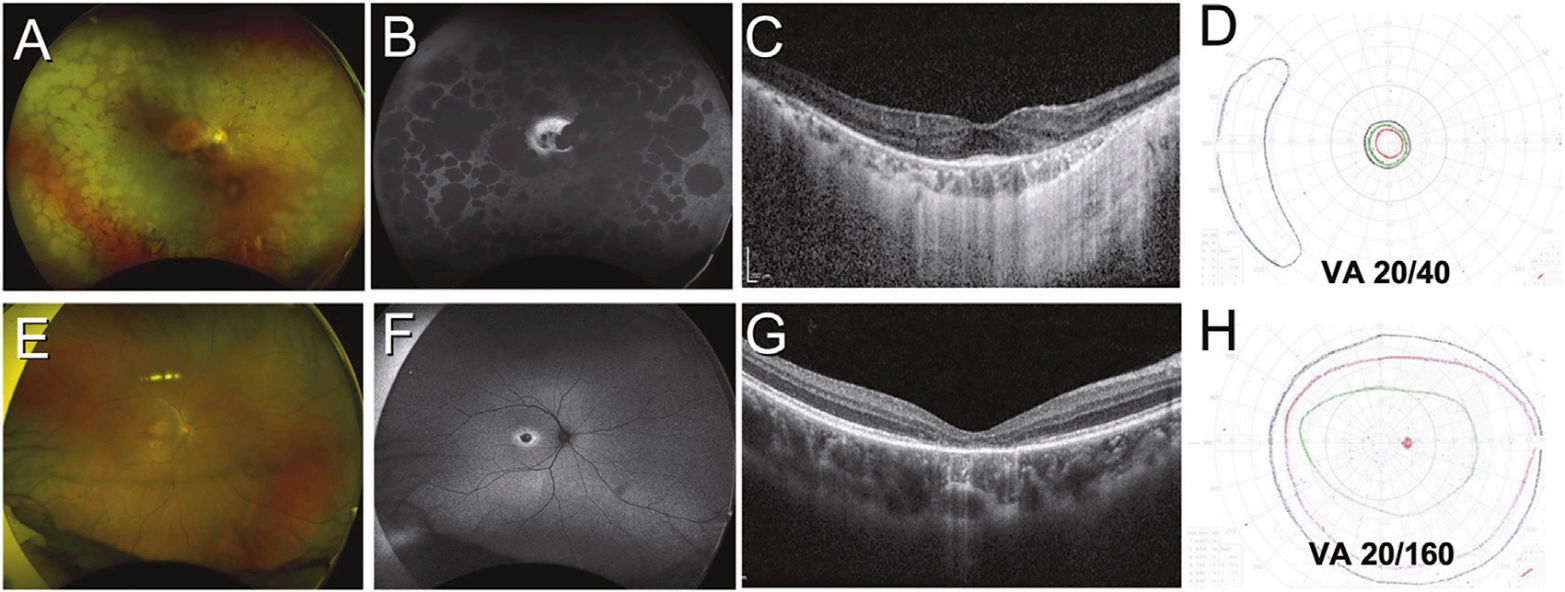
Variation in structural and functional changes due to inherited retinal disease. Case 1 **(A–D)** was a 51-year-old Indonesian male with retinitis pigmentosa secondary to *PRPH2* mutation. Retinal findings include diffuse peripheral RPE atrophy seen on color and autofluorescent imaging **(A,B)** with sparing of the central macula **(B)** and a small focus of foveal outer retina intact on OCT imaging **(C)**. Visual acuity was only mildly impaired, but the patient had tunnel vision as demonstrated by Goldmann kinetic perimetry **(D)**. Case 2 **(E–H)** was a 21-year-old Chinese male with cone dystrophy secondary to *PROM1* mutation. Retinal findings are limited to subtle pigmentary changes at the macula **(E)** which are highlighted on autofluorescent imaging **(F)**, while OCT reveals loss of the outer retina at the foveal region **(G)**. Visual fields are largely preserved but the patient had severely impaired visual acuity **(H)**.

In rod-cone dystrophies (RCD), or retinitis pigmentosa (RP), patients present with nyctalopia (night blindness), usually within the first or second decade of life. Presentation may be delayed depending on the environment, with patients living in well-lit urban settings often presenting later (Hartong et al., 2006). The second hallmark of RP is gradual, and often insidious, progressive loss of the peripheral visual field. This is seldom noticed by patients until it begins to encroach on the central vision. Field loss in RP patients has been reported to occur at a rate of approximately 5–10% per year (Berson et al., 1985), or 50% over 5 years (Massof et al., 1984). Importantly, in RP, like most IRDs, vision loss is generally symmetrical between two eyes. Central visual acuity can be affected early in the course of disease due to macular edema, which is to some extent treatable (Huckfeldt and Comander, 2017; Bakthavatchalam et al., 2018), or later in the disease as a result of cone involvement with loss of the photoreceptor-containing ellipsoid zone (EZ; Figure 1 and Figure 2). The presence of lyonization among female carriers of X-linked RP can cause asymmetrical disease expression between eyes, making diagnosis more challenging (Wuthisiri et al., 2013; Fahim et al., 2020).

In contrast to RCDs, patients with cone-rod dystrophies (CRDs) present with essentially the opposite sequence of symptoms. Visual acuity, impaired color vision, and photosensitivity occur first, followed later by visual field loss and nyctalopia (Figure 2). Predictably, CRDs result in more severe visual impairment earlier on in the course of the disease, although the end-point for both RCDs and CRDs is essentially the same (Hamel, 2007).

Both RCDs and CRDs appear to result in primary defects in rod and cone photoreceptors and animal models have been used to demonstrate that multiple points along the pathways of photoreceptor anabolism and catabolism can give rise to the phenotypes (Roosing et al., 2014; Sahel and Léveillard, 2018; Verbakel et al., 2018).

Inherited chorioretinal degenerations are a group of diseases characterized by early degeneration of the retinal pigment epithelium (RPE) that progresses to involve the choriocapillaris, Haller and Sattler layers of the choroid, and the photoreceptors in the later stages. The presentation of this subgroup of IRDs varies depending on the disease and may involve loss of central vision such as in the case of central areolar choroidal dystrophy (Boon et al., 2009) or progressive loss of peripheral vision in gyrate atrophy and choroideremia (Dimopoulos et al., 2017; Balfoort et al., 2021).

IRDs involving the macula, including Stargardt disease, Best disease, and pattern dystrophies among others, are more clinically heterogeneous than the preceding three subtypes and their diagnosis is usually more challenging because of their overlap with a multitude of acquired diseases. All share the clinical features of disease that is largely confined to the macula or posterior pole with symptoms affecting central vision, though the reasons for their predilection for the macula remains unclear. Current research suggests that differences in the structure of the choroid, Bruch’s membrane, RPE, photoreceptors, light exposure, and localized gene expression patterns may each play a part (Johnson et al., 2017; Tsang and Sharma, 2018).

## Measuring Outcomes in Inherited Retinal Diseases Therapy Trials

Before discussing specific gene-based therapeutic approaches for IRDs, it is instructive to review the approaches now being taken in clinical trials to assess the structural and functional outcomes of IRD treatments. Outcomes for common retinal diseases such as neovascular age-related macular degeneration (AMD) and diabetic macular edema (DME) have been relatively straightforward to assess, as the disease progresses on a time scale of months and treatment effects, such as that due to intravitreal anti-vascular endothelial growth factor (anti-VEGF), similarly take place over months or even weeks (Wells et al., 2016; Mitchell et al., 2018). Moreover, the dramatic functional and structural improvements seen after anti-VEGF therapy has meant that changes in visual acuity (measured in LogMAR letters) and direct imaging of the macula by optical coherence tomography (OCT) are typically sufficient endpoints for clinical trials of new drugs (Rofagha et al., 2013; Wong et al., 2016). Inherited retinal diseases, on the other hand, are far more insidious and cause vision loss over years and decades, with vision loss often being confined to the peripheral vision or loss of color vision, night vision, or contrast sensitivity in the early stages. Similarly, the structural changes cause by IRDs can initially be very subtle at disease onset, with easily quantifiable changes taking years to develop. These differences have made the assessment of clinical outcomes for IRD therapies particularly challenging, especially given the heavy financial investment and time constraints of for-profit pharmaceutical ventures. To address these limitations, clinical trials for IRDs often require multiple specialized functional and structural assessments to demonstrate treatment efficacy and these are briefly discussed here.

### Functional Assessment in Inherited Retinal Disease Treatment Trials

The best-corrected visual acuity (BCVA) achieved following manifest refraction, and the extent of a test subject’s visual field assessed with static or kinetic perimetry (Figure 2) remain clinical gold standards for functional outcome in IRD treatment trials. The selective loss of rod and/or cone function in many IRDs has prompted the development of specialized perimetry that can preferentially assess each cell type. In standard perimetry a white background is used with presentation to the subject of stimuli of increasing size and luminance to map the visual field. This approach predominantly tests long- and mid-wavelength-sensitive cones (LWC/MWS cones), while chromatic perimetry with monochromatic light has been used to highlight field deficits in cone diseases including achromatopsia and blue cone monochromacy (McGuigan et al., 2016; Bennett et al., 2017). Standard perimetry has been employed in several trials of IRD therapeutics (Schwartz et al., 2015; Maguire et al., 2019), with loss of central or peripheral field in treated eyes being compared with untreated eyes as a primary or secondary outcome. Although disease progression is slow, there is a demonstrable loss of field over a 1–2 years period in common forms of RP including *USH2A* disease (Xu et al., 2020).

In the majority of IRDs, including RCDs and CRDs, visual field assessment by perimetry is often not feasible as patients progress to perimetric blindness, tunnel vision, or have inadequate fixation for reliable assessment. This is especially true of the early-onset RCDs like LCA. To overcome this problem, clinical trials for IRDs have employed the full-field stimulus test (FST), which can evaluate visual function in patients with severe vision loss (Roman et al., 2021). FST measures the sensitivity of the entire visual field by providing an estimation of the lowest level of luminance that elicits a visual sensation by the subject. The FST presents stimuli of varying luminance according to a prespecified algorithm and the patient presses a button when a visual sensation is perceived. The FST instrument can additionally present varying colors to preferentially test different photoreceptor subsets, or be undertaken following dark adaptation to distinguish between cone and rod deficits (Roman et al., 2021; Tuohy and Megaw, 2021).

The transient pupillary light reflex (TPLR) is another objective measure of retinal function that remains intact even in severe and advanced IRDs (Cideciyan et al., 2021b). This response is typically measured with a high frame rate infrared video camera and specialized recording software under controlled lighting conditions with adjustable light stimuli (Kawasaki et al., 2012; Rukmini et al., 2019). Patients with advanced IRDs that affect both rods and cones display a TPLR reduction of more than 5 log units, offering excellent dynamic range (Rukmini et al., 2019; Cideciyan et al., 2021b) and showed treatment-dependent improvement in phase 1 trials of *RPE65* gene replacement therapy in LCA patients (Maguire et al., 2009).

Beyond specialized physiological testing, the need to demonstrate practical benefits for patients undergoing gene-based IRD therapies has driven the development of validated navigational assessment tools. The multi-luminance mobility test (MLMT), originally developed for use in an *RPE65* gene therapy trial (see below), requires patients to navigate a short obstacle course that simulates daily walking environments (Chung et al., 2018). MLMT scores are based on accuracy and speed, with a pass defined as completion of the course at a specified background luminance with less than four errors and within 3 mins (Russell et al., 2017; Chung et al., 2018).

### Structural Assessment in Inherited Retinal Diseases Treatment Trials

Contemporary structural assessment of the retina in IRDs is centred around the use of two core technologies: optical coherence tomography (OCT), and fundus autofluorescence (FAF). OCT is a non-invasive imaging modality that uses low coherence interferometry to quantify the intensity of light reflected from different structures within the retina. Current high resolution OCT, including spectral domain (SD-OCT) and swept-source (SS-OCT) instruments enable near-histology level resolution of retinal layers and enables both qualitative and quantitative assessment of outer retinal atrophy and photoreceptor loss in IRDs (Georgiou et al., 2020) (Figure 1).

Progressive outer retinal atrophy is a hallmark of the majority of IRDs and this can be readily visualized using FAF imaging. In conditions of high metabolic stress resulting from photoreceptor degeneration, there is an accumulation of fluorophores within the RPE, the predominant species being lipofuscin. FAF imaging enables visualization of these fluorophores as a bright hyperautofluorescent signal (Figure 1). Eventually the RPE and photoreceptors undergo apoptosis, and this signal is lost (Spaide, 2003; Sparrow and Boulton, 2005; Ach et al., 2015), leading to hypoautofluorescence (Figure 1). IRDs often display typical patterns on FAF imaging that are helpful in diagnosis, but the progression of the hyperautofluorescent signal (leading disease front), and the hypoautofluorescent signal (trailing disease front) can also be used to assess the rate of disease progression (Cicinelli et al., 2020; Daich Varela et al., 2021).

Adaptive optics (AO) is a novel imaging modality that measures wavefront aberrations from the retina to produce images of retinal cell layers with extremely high spatial resolution, sufficient to visualize individual photoreceptor cells (Akyol et al., 2021). This technique is non-invasive and has been used to characterize unique morphological changes that occur in various IRDs (Georgiou et al., 2018; Gill et al., 2019). It is expected that AO will become important for patient selection and disease monitoring in future clinical trials of IRD therapeutics.

## Approaches to Inherited Retinal Diseases Treatment

Over the past two to three decades numerous research groups worldwide have assembled and meticulously characterized the phenotypes and genotypes of large cohorts of IRD patients, with the largest reported cohorts numbering in the thousands (Gao et al., 2019; Pontikos et al., 2020; Sharon et al., 2020; Weisschuh et al., 2020; Qian et al., 2021). This has been accompanied by the development of cell and animal models of IRDs that have greatly improved our understanding of the molecular events that lead to disease expression for numerous gene variants. This, combined with the uniquely favorable features of the human eye, including ease of access, immune-privileged status, and the robust suite of noninvasive functional and structural investigations currently available, has resulted in IRDs being perceived as one of a limited number of ideal targets for the nascent field of gene-based therapy (Trapani and Auricchio, 2019; Hu et al., 2021). Despite this, the treatment of IRDs with targeted gene therapies presents numerous challenges to both the clinician and basic scientist. In the sections below we will review these challenges and provide examples of treatment approaches that aim to address each of them and lead to effective therapeutic interventions that will provide clinically meaningful benefits to IRD patients.

### Spectrum of Genes in Inherited Retinal Diseases

To date approximately 270 unique genes have been identified to be responsible for IRDs, with loci spanning all autosomes and both sex chromosomes. Despite this diversity and the widespread availability of high-throughput DNA sequencing, current cohort studies of IRD patients typically achieve only 50–70% hit rates in genotyping analysis (Van Schil et al., 2018), indicating that a substantial number of unidentified variants are responsible for the global IRD burden. Among known IRD genotypes, only a small number of genes are individually responsible for more than 1% of IRD cases in most published cohorts (Table 1). Among these, variants within *ABCA4* (9.3–20.8%), *EYS* (0.6–23.5%), *USH2A* (0.6–9.1%), *PRPH2* (0.4–4.6%), *RHO* (0.5–3.4%), and *RPGR* (1.2–5.7%) account for up to 50% of all successfully genotyped IRD cases across multiple ethnic cohorts (Table 1). Less common but responsible for one of the more severe IRD phenotypes seen in clinical practice is *RPE65*, whose mutation can cause an early onset RCD termed Leber congenital amaurosis (LCA). Inheritance of IRD genes can follow both recessive and dominant patterns (Daiger et al., 2014) and the size of the responsible genes varies enormously. This variability has important implications for gene-based therapies for IRDs.

**TABLE 1:** Contributions of selected genes to the IRD burden among different regional cohorts.

Contribution of selected gene mutations to IRD burden in study cohort								
Affected genes	Retinal phenotype	Germany (*n* = 1785)	Israel (*n* = 2,420)	Japan (*n* = 349)	Taiwan (*n* = 312)	United Kingdom (*n* = 3,195)	United States (*n* = 1,000)	Range
		Weisschuh et al. (2020)	Sharon et al. (2020)	Arai et al. (2015)	Chen et al. (2021)	Pontikos et al. (2020)	Stone et al. (2017)	
Study diagnostic rate (%)		70.8	56.0	45.6	57.1	N/A	76.0	
*ABCA4*	Stargardt disease, CRD	10.5	11.5	0.9	9.3	20.8	17.3	0.9–20.8
*BEST1*	Best disease	1.3	1.0	0	1.3	3.9	2.5	0–3.9
*CEP290*	LCA, RP	0.06	0.2	0	2.2	0.8	1.8	0–1.8
*CHM*	Choroideremia	2.3	0.7	0	0.6	2.7	1.4	0–2.7
*CRB1*	LCA, RP	1.4	1.3	1.4	1.3	2.1	2.0	1.3–2.1
*CYP4V2*	Bietti disease, RCD	0.1	0.2	2.0	3.8	0.6	0.0	0–3.8
*EYS*	RP	1.8	2.6	23.5	7.4	1.2	0.6	0.3–23.5
*GUCY2D*	LCA, RP, CRD	0.4	1.4	0.6	1.0	1.2	0.4	0.4–1.4
*PROM1*	Macular dystrophy, CRD, RP	1.2	0.1	1.2	1.6	1.2	0.6	0.1–1.6
*PRPF31*	RP	2.9	0.4	0	2.2	1.8	1.5	0–2.9
*PRPH2*	Pattern dystrophy, RP	2.7	0.7	4.6	1.0	4.6	3.2	0.7–4.6
*RDH12*	LCA, RP	0.4	1.3	0	1.0	1.1	0.6	0–1.3
*RHO*	RP, stationary night blindness	3.1	0.5	2.0	1.0	3.3	3.4	0.5–3.4
*RLBP1*	Retinitis punctata albescens	0.2	0.2	0.3	1.9	0.2	0.1	0.1–1.9
*RP1*	RP	1.0	0.3	0.3	1.3	3.3	1.0	0.3–3.3
*RPE65*	LCA, RP	0.3	0.8	0	1.6	1.2	0.3	0–1.6
*RPGR*	RP, CRD, cone dystrophy	5.7	1.6	1.2	2.6	5.1	4.8	1.6–5.7
*RS1*	X-linked retinoschisis	1.0	0.4	0	1.0	3.3	1.3	0–3.3
*USH2A*	RP	8.5	5.5	0.6	7.0	9.1	7.6	0.6–9.1

Among more than 300 inherited retinal disease entities caused by variations within more than 270 genes, approximately 70% are inherited in an autosomal recessive manner and 25% are autosomal dominant, with the remainder being X-linked or mitochondrial diseases (RetNet www.sph.uth.edu/retnet). Treatment of autosomal recessive disease is intuitive in the sense that replacement of the defective gene with a functional copy should ameliorate disease, and this approach has already been applied with some success in the case of, for example, *RPE65* mutations responsible for LCA (Russell et al., 2017). However, this approach is of limited value in autosomal dominant diseases where gain-of-function mutations are responsible for the disease phenotype. One of the most important causes of autosomal dominant RP is that caused by gain-of-function mutations in the rhodopsin gene, *RHO*, which is responsible for approximately 15% of all IRDs (Tsai et al., 2018). More than 100 such mutations in *RHO* are known and the unique challenges posed by these types of IRDs has prompted development of some novel therapeutic strategies which are discussed below.

Disease-causing variants of *ABCA4* (ATP binding cassette subfamily A member 4) are responsible for Stargardt disease and RP (Cremers et al., 2020), and in most characterized cohorts it is the single most prevalent gene responsible for IRDs. The *ABCA4* gene is just over 135 kbp in size, inclusive of noncoding regions, while the mature mRNA transcript is 7.3 kb (see Table 2). Variants of *EYS* (eyes shut homolog) and *USH2A* (usherin) are among the most frequently encountered genetic variants responsible for RP (Gao et al., 2019; Pontikos et al., 2020; Toualbi et al., 2020; Weisschuh et al., 2020; Yang et al., 2020; Qian et al., 2021). The *EYS* gene is 1.99 Mbp with a coding sequence length of 9.4 kbp, while the *USH2A* gene is 807 kbp with a coding sequence length of 15.6 kbp. On the smaller end of the gene spectrum, the *CYP4V2* (cytochrome P450 family 4 subfamily V member 2) gene responsible for Bietti crystalline retinal dystrophy (Astuti et al., 2015) and the *RPE65* (retinoid isomerohydrolase RPE65) gene responsible for LCA are both approximately 28 kbp with coding sequences of 1.6 kbp (Table 2). This variation in gene size is an important driver of the approaches taken to develop gene-based therapies and we have used this to provide a framework for our discussion below.

**TABLE 2:** Features of selected loci responsible for IRDs.

Gene	IRD phenotype	Chromosome location	Gene length (bp)	Coding sequence (bp)	Encoded protein	Protein function	Genbank accession
*ABCA4*	Stargardt disease	1	135,313	6,819	ATP binding cassette subfamily A member 4	Photoreceptor transport of all-trans-retinal aldehyde	NG_009073
*BEST*	Best disease	11	21,580	1,755	Bestrophin 1	Epithelial chloride ion channel	NG_009033
*CEP290*	LCA, RP	12	100,204	7,437	Centrosomal protein 290	Cilium formation	NG_008417
*CHM*	Choroideremia	X	193,383	1,959	CHM Rab escort protein	Rab GTPase	NG_009874
*CYP4V2*	Bietti crystalline dystrophy	4	28,939	1,575	Cytochrome P450 family 4 subfamily V member 2	Fatty acid precursor metabolism	NG_007,965
*EYS*	RP	6	1,994,246	9,432	Eyes shut homolog	Photoreceptor-specific, secreted matrix protein	NG_023443
*RHO*	RP, stationary night blindness	3	13,706	1,044	Rhodopsin	Rod-specific phototransducer	NG_009115
*RPE65*	LCA, RP	1	28,136	1,599	Retinoid isomerohydrolase RPE65	Isomerization step of 11-cis retinal synthesis	NG_008472
*USH2A*	RP, Usher syndrome II	1	807,558	15,606	Usherin	Photoreceptor and auditory hair cell maintenance	NG_009497

### Gene Replacement Strategies

**FIGURE 3:**
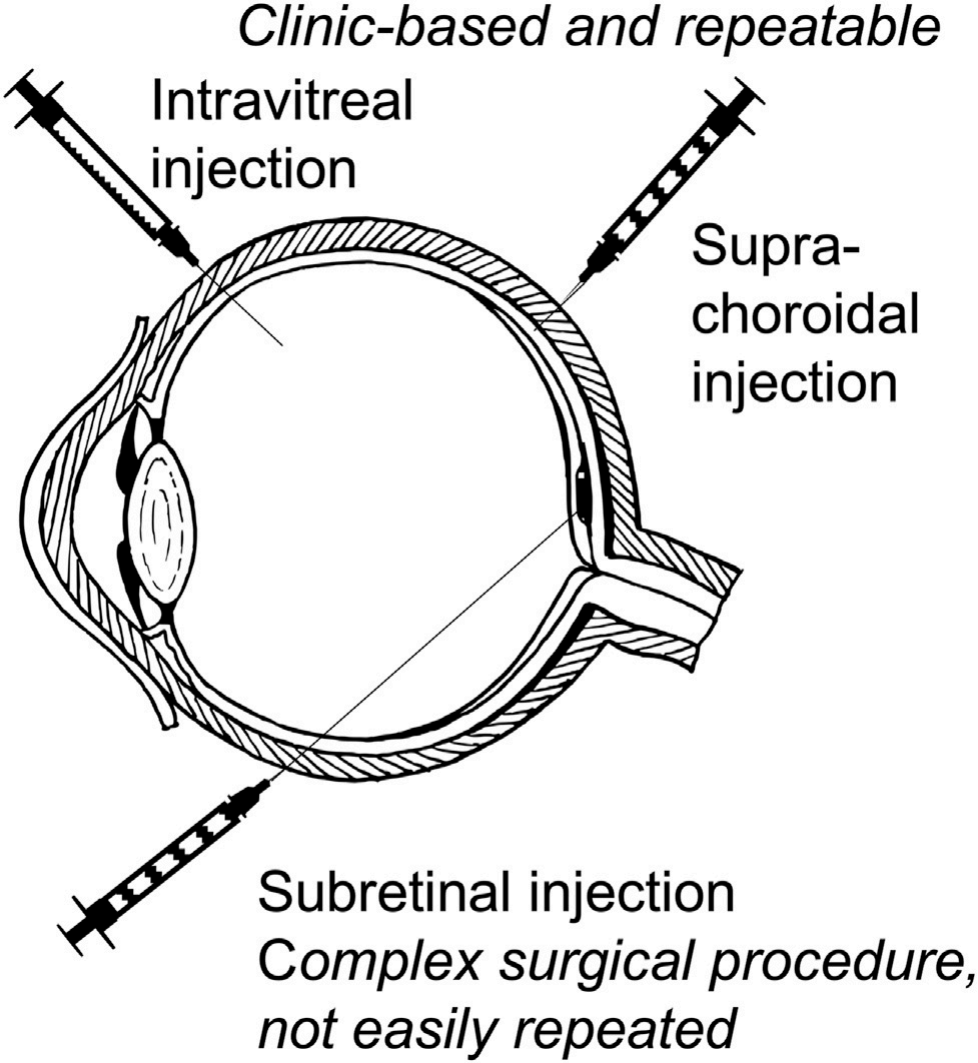
Approaches to retinal delivery of gene-based therapies for inherited retinal diseases. Intravitreal injection and the more recently developed suprachoroidal injection may be given as an outpatient clinic-based procedure with multiple repeat injections possible. Subretinal injection is a formal surgical procedure requiring pars plana vitrectomy and gas tamponade and is not easily repeated.

In its simplest implementation, gene replacement for IRDs aims to restore or maintain visual function by introducing a functional copy of a protein coding sequence into a target retinal cell population that is partially or completely deficient of the protein in question. In most cases this refers to biallelic autosomal recessive mutations which account for the majority of IRDs (Sahel et al., 2014; Carss et al., 2017). As a target tissue for gene replacement, the human retina has several advantages over other anatomical sites. Accessing the retina is relatively straightforward for the retinal specialist, and can be accomplished via intravitreal, subretinal, or suprachoroidal routes with outpatient-based surgical or procedural approaches (Figure 3). Additionally, blood-retinal barriers render the retina immune privileged, reducing the risk of immune reactions against gene delivery vectors. That said, given that the majority of IRDs cause pathology at the level of the photoreceptors or RPE (Figure 1), the overlying retinal cell layers including the inner and external limiting membranes form a barrier to the entry of vector particles larger than about 30 nm in size (Teo et al., 2018; Tavakoli et al., 2020), which limits the use of large vectors for intravitreal administration.

Current approaches to IRD gene replacement can be divided into viral and nonviral vectors. Several viruses have been investigated as potential vehicles for retinal gene replacement, including adenovirus, lentivirus, herpesvirus, and adeno-associated virus (AAV). The greater safety profile, lower inflammatory response, low incidence of host genome integration, and high efficacy of AAV has made it the vector of choice for retinal gene therapy (Hauswirth, 2014). AAV is a nonenveloped ssDNA parvovirus with a 25 nm particle size that under normal circumstances depends on co-infection by adenovirus or herpesvirus for replication (Balakrishnan and Jayandharan, 2014). The extraordinary number of AAV capsid variants has enabled the development of AAV vectors with a wide variety of host cell specificities, or tropisms. The most common recessive IRDs that can potentially benefit from gene replacement involve mutations in genes expressed mainly in photoreceptors and RPE (Figure 1). Less common IRDs such as congenital stationary night blindness (CSNB) may alternatively involve inner retinal layers (Zeitz et al., 2015).

Addressing the need to target one or several cell types within the retina has been an essential component of gene-based therapeutic development for IRDs. Work from the early 2000’s demonstrated that AAV serotypes displayed highly variable tropism for different human tissues (Wu et al., 2006; Srivastava, 2016; Li and Samulski, 2020). More recent research on AAVs for retinal targeting has highlighted the differences between AAV serotypes for transduction of the numerous cell populations within the human retina. By far the most extensively studied serotype of AAV is type 2 (AAV2), although recent work has demonstrated that the AAV2 viral capsid may not necessarily be the optimal subtype for transduction of all retinal cell types. Using a human retinal explant model, Wiley et al. (2018) demonstrated that AAV serotypes 1 (AAV1) and 4 (AAV4) appeared to have the highest intrinsic affinity for the outer nuclear layer (Table 3), which is where photoreceptor nuclei reside and is thus the preferred target for many IRD therapies. The same group subsequently demonstrated that gene delivery by AAV serotypes also varied based on the route of delivery, with intravitreal delivery resulting in low transduction efficacy with most serotypes and subretinal and suprachoroidal injection yielding high transduction efficacy only with AAV1 when using a mouse model for transgene delivery (Han et al., 2020). Further improvements to the native affinities of the AAV capsid for retinal cell types have been made by directed evolution approaches. The use of both *in vitro* and *in vivo* directed evolution has been used to dramatically improve the transduction of nonhuman primate photoreceptors following intravitreal delivery of the evolved AAV vectors (Dalkara et al., 2013; Byrne et al., 2020). Despite these improved vectors, however, only a relatively small percentage of all retinal photoreceptors are typically transduced even when high titers (>10^12^ virions) of AAV are injected (Dalkara et al., 2013).

**TABLE 3:** Variability in retinal cell transduction by different adeno-associated virus (AAV) capsid serotypes. Relative transduction efficiencies are shown for AAV capsid serotypes each carrying an AAV2 vector with a green fluorescent protein (GFP) gene under the control of a human cytomegalovirus (hCMV) promoter. These were used to transduce cadaveric human retinal explants. Data derived from Wiley et al. (2018).

AAV capsid serotype	Relative transduction efficiency in human retinal explants		
	ONL	INL	GCL and NFL
AAV1	High	Low	High
AAV2	Intermediate	Intermediate	High
AAV4	High	High	High
AAV5	Intermediate/high	Low	Low
AAV6	Intermediate/high	High	High
AAV8	Low	Intermediate	High
AAV9	Intermediate	Intermediate	High

The first FDA approved IRD gene therapy utilized AAV2 (Russell et al., 2017) to replace defective *RPE65* in patients with LCA. In this therapy the 1.6 kb coding sequence of *RPE65* was placed under the control of a modified avian *β*-actin promoter with a cytomegalovirus enhancer sequence (Maguire et al., 2008; Maguire et al., 2009). This cassette was inserted between AAV inverted terminal repeats (ITRs) and the vector propagated in HEK293 cell cultures. In the phase 3 trial of this vector, termed voretigene neparvovec (branded as Luxterna, Spark Therapeutics), LCA patients with biallelic *RPE65* mutations aged 3 and above and with visual acuity of 20/60 or worse or visual fields less than 20°, or both, underwent pars plana vitrectomy and subretinal injection of 0.3 ml (1.5 × 10^11^ vector genomes) of the vector (Russell et al., 2017). This approach had previously been demonstrated to yield efficient photoreceptor transduction in canines and nonhuman primates (Jacobson et al., 2006a; Jacobson et al., 2006b).

The primary endpoint for the phase 3 trial of voretigene neparvovec was patient performance on the abovementioned MLMT. At 1 year after treatment, the MLMT change score was 1.8 compared to 0.2 in the control group (*p* = 0.0013), with 65% of test subjects and no control subjects able to pass the MLMT at the lowest luminance of 1 lux (Russell et al., 2017). More recently, the three -year outcomes became available and showed a mean MLMT score of 2.4 among the test subjects, with 71% able to pass the MLMT at the lowest luminance, with visual acuity remaining essentially unchanged (Maguire et al., 2021). The most notable complication among the test subjects was a retinal detachment that occurred in a single patient (1 of 21, 4.8%) at around year four, which was probably related to the original surgical intervention and is a known complication of routine vitrectomy. Additional complications, including endophthalmitis, development of macular holes, raised intraocular pressure, macular edema, and macular atrophy can occur following vitrectomy and subretinal injection (Nuzbrokh et al., 2020), and larger cohort studies of treated IRD patients will be required before their incidence can be properly assessed.

Despite the numerous advantages of AAV for retinal gene delivery, its small size places limits on its cargo capacity. The wild-type AAV genome is 4.8 kb in size and contains short 5ʹ and 3ʹ ITRs that flank coding sequences for replication (Rep) and capsid (Cap) proteins (Puppo et al., 2014). Modified cell lines that express Rep and Cap enable for this entire central region to be replaced by an insert size of up to 4.4 kb. For gene replacement therapy this insert needs to include both the protein coding sequence as well as well as upstream and downstream regulatory elements to ensure efficient and appropriate expression of the IRD transgene. Given the limited cargo capacity of AAV and the prevalence of IRDs caused by large gene variants such as *USH2A* (15.6 kb) and *EYS* (9.4 kb), researchers have sought to engineer AAV-based vectors that can deliver larger genes. Dual AAV systems enable a near-doubling of deliverable transgene size by dividing the transgene into halves and appending overlapping sequences to the termini to enable homologous recombination (Trapani, 2018). Alternatively, a splicing acceptor and splice donor can be added to the termini to enable intermolecular concatamerization and splicing of the two halves into a single contiguous cDNA construct (Carvalho et al., 2017). *In vivo* efficacy of a dual AAV vector system has previously been demonstrated in a mouse model of Stargardt disease (McClements et al., 2019). Two fragments of the 6.8 kb *ABCA4* coding sequence, 3.7 and 3.3 kb in size and with 207 bases of overlapping sequence were used to develop two vectors, with the upstream fragment being driven by a rhodopsin kinase promoter and the downstream fragment terminated by a hepatitis virus post-transcriptional regulatory element (McClements et al., 2019). Subretinal co-delivery of these constructs yielded robust full-length *ABCA4* expression in photoreceptor outer segments and successfully reduced accumulation of toxic *bis*-retinoids that accumulate in the *ABCA4*-deficient retina. Human clinical trials involving the use of dual AAV systems are in the planning stage but have yet to be initiated (Piotter et al., 2021).

Nonviral approaches to IRD gene replacement have been explored with the aim of overcoming the gene size limitation of AAV in addition to improving the safety profile and production costs associated with viral vector-based approaches. Synthetic vectors, or nanoparticles, are nonviral vectors comprising a cationic lipid assembly that can encapsulate a transgene of interest up to 20 kb in size and deliver it through nuclear pores to enable gene expression. A variety of nanoparticle materials have been developed and contemporary nanoparticles can transfect both RPE and photoreceptors, albeit with less proven durability than that seen after AAV2 transduction with IRD-related genes (Conley and Naash, 2010; Adijanto and Naash, 2015; Trigueros et al., 2019; Sun et al., 2020).

### Genome Editing Strategies

Despite being the predominant treatment modality in current IRD clinical trials (Prado et al., 2020), conventional gene augmentation therapy is limited to the treatment of loss of function genotypes and haploinsufficiency, with no direct modification of the host genome. Moreover, contemporary gene augmentation vectors with demonstrated efficacy for *in vivo* transduction of retinal photoreceptors are limited in their maximum cargo size (Puppo et al., 2014). This renders gene augmentation unsuitable for many important IRD genotypes, such as RP due to *USH2A* and *EYS* variants. Gene editing approaches aim to address these limitations by correcting disease-causing mutations at the level of the host genome.

Human genome editing was originally made possible due to pioneering work in the early 2010’s that demonstrated the use of a clustered regularly interspaced short palindromic repeat (CRISPR) and Cas9 nuclease (CRISPR/Cas9) system derived from *Streptococcus pyogenes* to introduce site-specific nucleotide alterations in the target genome (Ran et al., 2013; Jiang and Doudna, 2017). In its simplest implementation, the CRISPR/Cas9 system works via a four-step process whereby: 1) the Cas9 protein forms a complex with a sequence-specific guide RNA within the cell; 2) the Cas9-RNA complex anneals to the complementary gDNA sequence; 3) the Cas9-RNA complex creates a double-stranded DNA break in the gDNA; and 4) a modification is made to the gDNA via endogenous DNA repair mechanisms—either homology-directed repair (HDR) or non-homologous end-joining (NHEJ) (Ran et al., 2013). The introduction of exogenous guide DNA enables the selective correction of deleterious mutations via HDR, while NHEJ creates errors at the target site and can selectively inactivate genes (Zhang, 2021).

At present the most important limitation of the CRISPR/Cas9 system is the introduction of unintended mutations at genomic locations containing sequence homology to the target site, also known as off-target editing (Zhang et al., 2015). Several approaches have been taken to overcome this problem for IRD gene editing, including the use of retinal cell-specific promoters to drive the expression of Cas9, directed mutagenesis of the Cas9 nuclease to improve its on-target/off-target editing and reduce its biological half-life, and the use of additional guide RNAs complexed to Cas9 to improve gene targeting (Burnight et al., 2018; Peddle et al., 2020). A second limitation of CRISPR/Cas9 gene editing for IRDs is the efficient delivery of the Cas9 expression cassette and guide RNA into the target retinal cell type. Numerous approaches have been described, including AAV-based vectors that utilize small Cas9 orthologs from other bacterial species (Gasiunas et al., 2020) and dual AAV systems that deliver the different elements of the Cas9, guide RNA, and donor DNA cassettes (Hung et al., 2018).

After several years of intensive basic and pre-clinical research on CRISPR/Cas9-mediated gDNA editing in IRD cell and animal models of disease (Maeder et al., 2019), this approach has now reached clinical trials. LCA type 10 (LCA10), a severe and early-onset IRD caused by bi-allelic loss-of-function mutations in the *CEP290* gene (coding sequence size of 7.9 kb). This 7.9 kb coding sequence of *CEP290* exceeds AAV insert capacity and gDNA editing, among other approaches, was seen as a viable therapeutic option. The most prevalent *CEP290* mutation causing LCA10 is IVS26, which introduces a premature stop codon via alterations to RNA splicing (Burnight et al., 2017). In the phase 1/2 EDIT-101 trial an AAV5 vector is used to deliver SaCas9 (from *Staphylococcus aureus*) and *CEP290*-specific guide RNAs to photoreceptor cells by subretinal injection. Wild-type *CEP290* mRNA is produced via intronic inversion or deletion mediated by the editing construct. In the case of LCA10, a minimum gDNA editing efficiency of 10% was determined in earlier studies to be required for meaningful vision restoration, and this baseline efficiency was exceeded in mouse and nonhuman primate models (Maeder et al., 2019). Final results from the EDIT-101 trial for LCA10 are expected in 2024, although initial clinical data from the phase 1/2 BRILLIANCE trial (Editas Medicine, Cambridge, Mass.) showed a positive safety profile at 15 months after treatment and limited evidence of clinical efficacy.

Genome editing is also being applied to the treatment of autosomal dominant IRDs, where a point mutation can result in production of a gain-of-function protein that impairs cell functions (Farrar et al., 2012; Athanasiou et al., 2018). Autosomal dominant RP caused by such mutations in *RHO* has received significant attention due to their relative prevalence in the IRD patient population. An emerging approach for the treatment of these *RHO* variants involves simultaneous ablation of the mutant *RHO* allele and replacement with wild-type *RHO* (Meng et al., 2020). Work by Tsai et al. (2018) demonstrated the use of a pair of AAV2/8 vectors to deliver either 1) guide RNAs that targeted the genomic DNA region up- and downstream of *RHO* start codon, in addition to a wild-type *RHO* expression cassette, or 2) a Cas9 expression cassette. The transduced version of the *RHO* gene resistant to CRISPR/Cas9 ablation complemented the mutant copy, while the endogenous mutated *RHO* was selectively targeted for ablation using cell and mouse models. This elegant approach ensures that ablation of the dominant mutant *RHO* gene will only occur in the presence of transduced wild-type *RHO*. Importantly, this approach is largely agnostic to the *RHO* mutation responsible for the phenotype. Related approaches that target specific *RHO* variants using CRISPR/Cas9 approaches were also described recently (Patrizi et al., 2021).

### RNA Editing to Treat Inherited Retinal Diseases

Genomic DNA editing has the potential to address the root cause of essentially all IRDs but concerns regarding off target editing and its potentially deleterious impacts on the eye (Smith et al., 2017) has prompted exploration of alternative methods to correct disease-causing variants. RNA editing is a normal biological process that occurs in human cells, including the retina, and can be harnessed to create sequence-specific nucleotide edits of mRNA. In its natural role, RNA editing is performed by adenosine deaminases (adenosine deaminase acting on RNA, or ADAR) and cytidine deaminases (cytidine deaminase acting on RNA, or CDAR) that can catalyze adenosine-to-inosine (A-I) and cytosine-to-uridine (C-U) deamination, which is functionally equivalent to A-to-G and C-to-T editing, respectively (Nishikura, 2010; Gallo et al., 2017). Because these edits can be targeted to a specific mRNA and do not affect genomic DNA, their impact is transient, as would be any potential off-target effects.

Humans possess two ADAR enzymes, ADAR1 and ADAR2, with the former being expressed throughout the retina and the latter being expressed mainly in retinal ganglion cells (Fry et al., 2020). Both enzymes are capable of editing mRNA and the process occurs in the nucleus concurrently with RNA splicing (Eisenberg, 2021).

In its most basic form, the sequence-specific A-to-I editing activity of ADAR requires two components: 1) a guide RNA (specificity domain), analogous to that used in the CRISPR-Cas9 system, which can anneal to the target mRNA, and 2) an ADAR recruiting domain that adopts a dsRNA hairpin structure and promotes ADAR recruitment to the targeted mRNA complex (Eisenberg, 2021). This approach enables the use of endogenous ADAR from host retinal cells, thus avoiding the need to introduce exogenous ADAR expression cassettes like that required for Cas9 (although this approach has been explored for ADAR). Instead, RNA editing can be accomplished with the use of custom antisense oligonucleotides (ASOs) that incorporate the specificity and ADAR recruiting domains, potentially simplifying the development of IRD genotype-specific treatments (Merkle et al., 2019). This ASO-only approach to RNA editing was successfully demonstrated in a variety of human cell lines by Merkle and others (Merkle et al., 2019) and achieved sequence-specific mRNA editing efficacy of 30–70% with endogenously expressed ADAR. Additionally, this approach incorporated the use of chemical modifications to the ASO (2′-O-methyl and phosphorothioate linkages) to improve their biological stability. Such an approach, even with the use of chemically stabilized ASOs would, however, require repeat dosing with the ASO over a patient’s lifetime to maintain efficacy.

The application of RNA editing to IRD treatment is presently limited by editing efficiency and potentially by off-target effects, and currently available tools appear to be primarily suited to the treatment of IRDs caused by a subset of recessive mutations within large genes that are not amenable to AAV-mediated gene augmentation. A recent survey by Fry and coworkers (Fry et al., 2020) found substantial heterogeneity in the proportion of IRD mutations amenable to RNA editing, with 9% of known pathogenic mutations in *CEP290* and 32% in *ABCA4* being correctable by RNA editing. At present it is unknown what level of RNA editing efficiency will be required to achieve biologically or clinically meaningful improvements in the wide variety of IRDs to which the technique is applicable, although the past several years have seen dramatic improvements in the range of RNA editing tools available for the creation of mutation-specific therapeutics and several industry-led trials of RNA editing for IRD treatment are expected to commence in the coming years.

### Antisense Oligonucleotides and RNA Interference for Gene Modulation in Inherited Retinal Disease

Antisense oligonucleotides (ASOs) are small single-stranded RNA or DNA sequences, typically in the range of 15–30 bases, that can be designed to anneal to specific mRNAs and effect gene silencing, inhibit protein translation, or alter mRNA splicing (Crooke et al., 2017; Bennett, 2019). This makes ASOs applicable to dominantly inherited gain-of-function alleles and recessive alleles with splicing defects. Upon annealing to their mRNA target, ASOs can direct RNase H-mediated mRNA cleavage or induce changes in RNA splicing that result in exon-skipping with shortening of the protein product, or nonsense-mediated decay of the mRNA (Maruyama and Yokota, 2020).

This class of IRD therapeutics is particularly attractive because they can be delivered intravitreally and penetrate the outer retinal layers, have an excellent safety profile, and can be produced at scale far more economically than other gene-based therapeutics (Chi et al., 2017; Xue and MacLaren, 2020). Moreover, initial problems with ASO stability *in vivo* have now been overcome with the development of nuclease-resistant phosphorodiamidate morpholino oligonucleotides (PMOs) that also have dramatically improved cell penetration compared to unmodified ASOs (Smith and Zain, 2019).

Clinical application of ASOs for IRD treatment has recently been demonstrated in several phase I/II clinical trials. An intronic mutation of *CEP290* (c.2991 + 1655A > G, or p. Cys998X) is a common cause of LCA10 and results in creation of a cryptic splice donor site, leading to a new exon and in-frame stop codon between the native exons 26 and 27 (Ramsbottom et al., 2018). A 17-mer modified ssRNA, termed Sepofarsen (ProQR Therapeutics, Netherlands), anneals to the mutated *CEP290* mRNA splicing site and is effective in restoring normal CEP290 function in cell and animal models (Barny et al., 2018; Ramsbottom et al., 2018). More recently, a phase I/II trial demonstrated rapid and sustained improvements in visual acuity, visual fields, electrophysiological parameters, and retinal structure following a single intravitreal injection of 160 μg or 320 μg of sepofarsen in *CEP290* p. Cys998X patients (Cideciyan et al., 2021a). A phase II/III trial of sepofarsen is currently ongoing.

Pathological variants of the *USH2A* gene, which encodes the usherin protein, are the most common cause of autosomal recessive syndromic and nonsyndromic RP worldwide and among the most common causes of congenital deafness (Toualbi et al., 2020). The c.2299delG and c.2276G > T mutations within exon 13 of *USH2A* are responsible for up to a third of RP cases in some populations (Pendse et al., 2019). These variants create a premature stop codon and lead to nonfunctional usherin, although removal of the exon 13 equivalent region from mouse *USH2A* did not appear to interfere with its biological function in a mouse model (Pendse et al., 2019). Moreover, the introduction of a morpholino ASO (QR-421a) that targets exon 13 and promotes exon skipping of this region was able to restore retinal function in zebrafish and mouse disease models (Dulla et al., 2021). The phase I/II STELLAR trial of QR-421a (ProQR Therapeutics, Netherlands) for *USH2A* exon 13-related RP showed retention of 1–2 lines of visual acuity in eyes treated with a single intravitreal injection of QR-421a, compared to the untreated fellow eyes.

These encouraging early clinical trial findings and the potential convenience with which new targeted ASOs can be developed and manufactured suggests they may play a role in treating not only the more prevalent IRDs like *USH2A*-related RP, but also rarer IRDs that might not otherwise be considered as viable therapeutic targets due to profit considerations by pharmaceutical companies.

Autosomal dominant IRDs are widely considered a key target for the use of gene knockdown approaches (Farrar et al., 2012; Meng et al., 2020). One promising gene knockdown approach for IRDs is RNA interference (RNAi), an important naturally occurring mechanism of gene suppression in eukaryotes. RNAi is driven by the production of small (average of 22-mer) RNAs, termed microRNAs (miRNAs), that are typically transcribed from the 5ʹ ends of mRNA and can selectively trigger mRNA decay in a sequence-specific fashion (O'Brien et al., 2018). MicroRNA precursor derivatives termed mirtrons are spliced from the 5ʹ end of mRNAs and can similarly knock down target mRNA with high specificity. (Okamura et al., 2007; Ruby et al., 2007). Conveniently, mirtrons can be incorporated into polycistronic expression cassettes that contain both the mirtron and an engineered copy of the target gene that is resistant to mirtron-mediated RNAi, making them particularly useful for autosomal dominant diseases. This approach has been demonstrated in a mouse model of *RHO* mediated RP, where a single AAV vector containing a mirtron that targeted both the wild-type and mutant copies of *RHO* for RNAi-mediated decay, and at the same time supplied a RNAi-resistant engineered copy of functional *RHO* to the transfected cell (Orlans et al., 2021). This mutation-agnostic approach may theoretically be applied to many dominantly inherited IRDs.

### Optogenetics as a General-Purpose Therapy for Late Stage Inherited Retinal Disease

Each of the gene based IRD therapies discussed above are limited in their application to patients with a specifically affected gene or gene variant. Given that IRDs are orphan diseases, it is expected that there will only be a relatively small number of individuals in any given population for whom such custom gene therapies are applicable. An important challenge in IRD therapy is thus the development of general-purpose therapies that can be used for a wide variety of IRDs irrespective of the patient’s particular genotype. One such general-purpose therapy approach which is still within the scope of gene-based therapy is optogenetics, which aims to restore vision in late-stage IRDs by targeting genes encoding photosensitive proteins to selected retinal cell types, converting them into a replacement photoreceptor (Duebel et al., 2015; McClements et al., 2020).

Optogenetic techniques were originally developed as research tools to explore the function of mammalian neurons in animal models (Adamantidis et al., 2007; Deisseroth, 2011; Zhang et al., 2011). In this technique, microbially-derived opsin proteins are used to trigger neuron firing in response to specific wavelengths of light (Williams and Deisseroth, 2013). Opsin genes can be inserted into a gene expression cassette and delivered *via* a viral vector into neurons *in vivo*, after which the transduced neurons are rendered photosensitive. In many IRDs, including RCDs and CRDs, there is loss of photoreceptors in the outer retina but relative preservation of the inner retinal layers, including the retinal ganglion cell (RGC) layer that coalesces to form the optic nerve head. The greater exposure of this retinal layer to the vitreous cavity, compared to the much deeper photoreceptor layer, makes it an attractive target for virus-mediated gene transduction. Proof-of-principle for optogenetic therapy was provided by Bi and others (Bi et al., 2006), who demonstrated successful phototransduction by bacterial channelrhodopsin (ChR2) in transduced mouse RGCs with signal propagation to the visual cortex.

More recently, optogenetics has been successfully translated into a general purpose IRD therapy. In groundbreaking work by Sahel and others (Sahel et al., 2021), a patient with late-stage RP and visual acuity of perception of light underwent intravitreal injection with an AAV containing an optogenetic expression cassette (AAV2.7m8-CAG-ChrimsonR-tdTomato). Following treatment, the patient underwent training with light-stimulating goggles that converted an external video feed into a pixel map projected onto a 10° circular area of the central retina using a diode light source specific for the optogenetic construct (ChrimsonR, peak wavelength of 595 nm). While the untreated eye remained at baseline vision, the treated eye gained the ability to perceive, locate, and count various objects whilst using the goggles (Sahel et al., 2021). The phase 1/2a PIONEER study will report on the findings of this approach in a small patient cohort, and the primary outcomes are expected to be available in the coming months. Promising early results from this trial have more recently enabled fast track status of this treatment (GS030, from GenSight Biologics, Paris) by the US FDA (FDA, October 2021).

**FIGURE 4:**
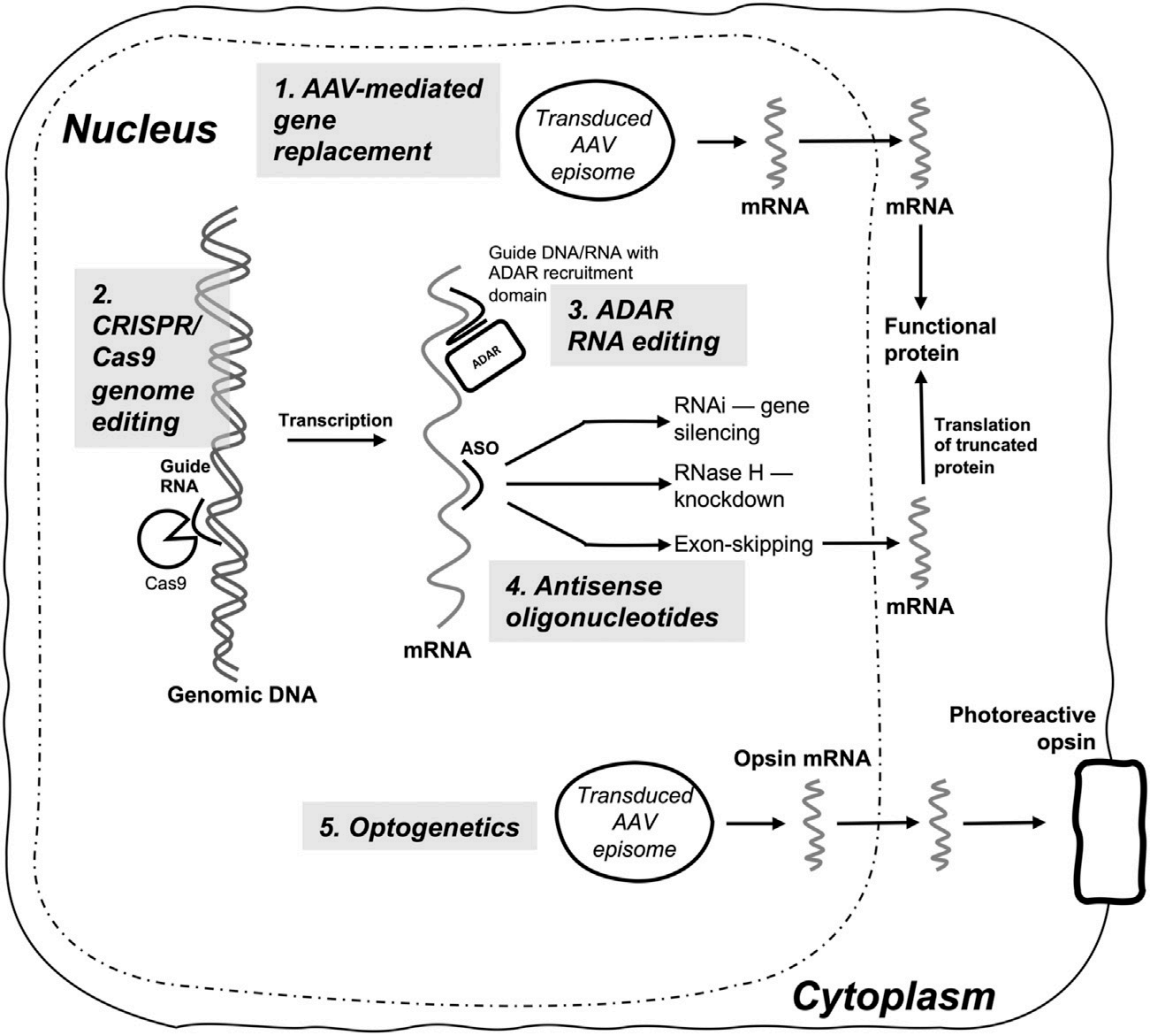
Summary diagram of gene-based therapies currently in clinical use or clinical trials for inherited retinal diseases. (1) AAV-mediated gene replacement therapy is currently the predominant modality, and delivers replacement transgenes (e.g., *RPE65*), or transgene fragments (dual AAV systems) to produce a functional protein in biallelic autosomal recessive IRDs; (2) CRISPR/Cas9 genome editing requires delivery of CRISPR/Cas9 constructs, most commonly with AAV vector systems, and enables site-directed editing or mutagenesis of IRD target genes (e.g., *CEP290*); (3) ADAR-mediated RNA editing is used to perform sequence-specific RNA nucleotide edits by utilizing guide DNA or RNA and an ADAR recruitment domain, with either endogenous ADAR or transduced and overexpressed exogenous ADAR enzyme; (4) Antisense oligonucleotides (ASOs) induce sequence specific gene silencing *via* RNAi, RNase H-mediated mRNA knockdown, or targeted exon skipping (e.g., *USH2A* exon 13); (5) Optogenetics delivers an engineered phototransducing opsin to a specific retinal cell type (e.g., ganglion cells) to render the cell photosensitive and capable of replacing the light-responsive function of degenerating photoreceptor cells in IRDs.

Nonselective optogenetic approaches using RGC transduction are likely sufficient to restore light sensitivity and gross visual function in IRD patients but do not enable image processing by retinal interneurons (Duebel et al., 2015). This places a limit on the quality of vision possible using the approach and for now the method is likely applicable mainly to individuals with severe vision loss. More selective approaches that involve targeted transduction or gene expression in retinal bipolar cells may enable more physiological activation of RGCs and potentially better restoration of visual quality (Gilhooley et al., 2021). An overview of optogenetic and other approaches to gene-based therapy for IRD treatment is shown in Figure 4.

## Conclusion

From the conception of human gene-based therapeutics in the middle of the last century there has been an increasing expectation by many in the medical and research community that, given a critical mass of knowledge and technology, it will eventually be possible to effectively cure genetic diseases (Miller, 1992; Wirth et al., 2013; Dunbar et al., 2018). IRDs are also seen as one of the lowest hanging fruits in this area, given their well-defined and typically monogenic basis and the sophistication of current molecular and surgical techniques. Additionally, the orphan status of IRDs typically facilitates faster regulatory approval and can improve early patient access to treatment (Haffner, 2005). Despite this, recent clinical trials have made it obvious that gene-based therapies still have a host of challenges that must be overcome if IRDs are to become a curable or at least manageable disease. Questions regarding the long-term safety and durability of AAV-based subretinal vector delivery remain, with long term follow up of patients treated with voretigene neparvovec-showing possible safety signals and a suggestion of visual acuity decline after 4 years (Maguire et al., 2021). While the current IRD clinical trial landscape remains dominated by AAV-based approaches requiring subretinal injection, an increasing number of conveniently administered intravitreal therapeutics such as ASOs are being trialed with encouraging early results.

The recent successful trial of genotype-agnostic optogenetic therapy for advanced RP (Sahel et al., 2021) prompts re-evaluation of the value of genotype-specific therapies. Given the large genotypic diversity of IRDs and the high development costs of current therapeutics, it is unlikely that custom gene-based therapies will be developed for all IRD mutations. While optogenetics may afford some level of visual improvement for subjects with advanced IRDs, its current implementation affords only limited visual improvement due to the complexity of retinal neuronal circuitry. General purpose cell-based therapeutics could potentially be applied to any degenerative condition of the outer retina, including IRDs, but have yet reach their potential in clinical trials for IRD patients (Ben M'Barek et al., 2019; Maeda et al., 2019). That said, we envisage general-purpose cell-based therapies and combination therapies involving cell-based and gene-based therapies to become much more dominant in the longer term.

Meanwhile, the recent proliferation of clinical trials for a host of gene based IRD treatments is providing invaluable data that will likely enable a small number of highly efficacious treatment approaches to be applied to the majority of IRDs. Despite many initial setbacks that have arisen in early trials it is becoming increasingly clear that the coming years will be pivotal learning experiences that will pave the way not only for IRD gene therapy, but for the more common and more genetically complex eye diseases.
